# Childhood exposure to parental smoking and life-course overweight and central obesity

**DOI:** 10.1080/07853890.2020.1853215

**Published:** 2021-02-22

**Authors:** Johanna M. Jaakkola, Suvi P. Rovio, Katja Pahkala, Jorma Viikari, Tapani Rönnemaa, Antti Jula, Harri Niinikoski, Juha Mykkänen, Markus Juonala, Nina Hutri-Kähönen, Mika Kähönen, Terho Lehtimäki, Olli T. Raitakari

**Affiliations:** a Research Centre of Applied and Preventive Cardiovascular Medicine, University of Turku, Turku, Finland; b Centre for Population Health Research, University of Turku and Turku University Hospital, Turku, Finland; c Paavo Nurmi Centre, Sports and Exercise Medicine Unit, Department of Health and Physical activity, University of Turku, Turku, Finland; d Department of Medicine, University of Turku, Turku, Finland; e Division of Medicine, Turku University Hospital, Turku, Finland; f Department of Chronic Disease Prevention, Institute for Health and Welfare, Turku, Finland; g Department of Pediatrics, Turku University Hospital, Turku, Finland; h Department of Pediatrics, Tampere University Hospital, and Faculty of Medicine and Health Technology, Tampere University, Tampere, Finland; i Department of Clinical Physiology, Tampere University Hospital and Faculty of Medicine and Health Technology, Tampere University, Tampere, Finland; j Department of Clinical Chemistry, Fimlab Laboratories and Finnish Cardiovascular Research Center-Tampere, Faculty of Medicine and Health Technology, Tampere University, Tampere, Finland; k Department Clinical Physiology and Nuclear Medicine, Turku University Hospital, Turku, Finland

**Keywords:** Smoking exposure, parental, overweight, central obesity

## Abstract

**Objective:**

To evaluate the association between childhood parental smoking exposure and the risk of overweight/obesity from childhood to adulthood.

**Methods:**

This study leverages the data from two longitudinal population based cohort studies, the Cardiovascular Risk in Young Finns Study between years 1980–2011/2012 (YFS; *N* = 2,303; baseline age 3–18 years) and the Special Turku Coronary Risk Factor Intervention Project between years 1989–2009/2010 (STRIP; *N* = 632; baseline age 7 months). Weight, height and waist circumference were measured from childhood to adulthood. Overweight/obesity was defined as body mass index ≥25 kg/m^2^ in adults and using the Cole criteria in children. Central obesity was defined as waist circumference > 100/90 cm in men/women and as a waist-to-height ratio > 0.50 in children. Statistical analyses were adjusted for age, sex, socioeconomic status, smoking, birth weight, parental ages, diet and physical activity.

**Results:**

Childhood parental smoking exposure was associated with increased risk for life-course overweight/obesity (YFS: RR1.13, 95%CI 1.02–1.24; STRIP: RR1.57, 95%CI 1.10–2.26) and central obesity (YFS: RR1.18, 95%CI 1.01–1.38; STRIP: RR1.45, 95%CI 0.98–2.15).

**Conclusions:**

Childhood exposure to parental smoking is associated with increased risk of overweight/obesity over the life-course.

**KEY MESSAGES**
- Exposure to parental smoking in childhood was associated with increased risk of overweight/obesity, central obesity and adiposity measured by skinfold thickness from childhood to adulthood.

## Introduction

Overweight and obesity have more than doubled since 1980 contributing to the aetiology of non-communicable diseases such as cardiovascular diseases [1]. Currently, from the adult Finnish population (age >30 years) 72% of men and 63% of women are overweight or obese [2]. Overweight usuallystarts to develop at an early age and it often also persiststo adulthood. New tools are needed to suppress the epidemic of overweight and obesitystarting from childhood.

Using the data from the Cardiovascular Risk in Young Finns Study (YFS) and Childhood Determinants of Adult Health, we have previously shown that childhood exposure to parental smoking is associated with decreased brachial flow-mediated dilatation [3], increased risk of carotid atherosclerosis plaque [4], increased carotid intima media thickness [5] and impaired bone mineral density [6] in adulthood. Together these findings suggest that second-hand exposure to parental smoking from childhood might have wide ramifications on the offspring’s health in adulthood.

The association of passive smoking during prenatal period on anthropometric measurements, including body mass index (BMI), waist circumference, central adiposity or adverse body and abdominal fat distribution in childhood [7–13] and adulthood [14] is well-known. Some evidence also suggest that the link between increased BMI among offspring of those parents who have begun smoking already in their childhood might act *via* epigenetic mechanisms [15–17]. In previous observational studies, early postnatal exposure to maternal smoking has been observed to increase offspring BMI in childhood [10,18,19] and central adiposity in adolescence [20]. In addition to the short-term associations observed in humans, animal studies have shown long-term effects of postnatal smoking exposure on BMI [21,22] as specifically smoking exposure during suckling has been suggested to increase BMI in adult rats.

To our knowledge there are no prior data on the associations of childhood passive smoking exposure with longitudinal obesity related anthropometric measurements in humans. Therefore, we aimed to close this gap of knowledge by exploring the associations of parental smoking exposure during childhood and the prevalence of overweight/obesity from early childhood to adulthood using the data from two longitudinal population-based studies, namely the YFS and the Special Turku Coronary Risk Factor Intervention Project (STRIP).

## Materials and methods

### Study designs and setting

This study leverages the data from two longitudinal population-based Finnish studies the YFS [23] and the STRIP [24]. In the YFS, the first cross-sectional survey was conducted in 1980, when 3596 randomly selected children and adolescents (boys and girls; aged 3, 6, 9, 12, 15 and 18 years) were recruited from the Finnish cities of Helsinki, Turku, Tampere, Oulu and Kuopio in addition to their rural surroundings. The cohort has been regularly followed-up at 3–9 years intervals. The latest follow-up study was carried out in 2011 (Supplementary Figure 1). The STRIP is an infancy-onset dietary intervention trial that was launched in 1989 in Turku (Supplementary Figure 2). Recruitment of the children and their parents was done at well-baby clinics during a 5-month-visit. The intervention and data collection were conducted at least annually until the age of 20 years (2011). In the present study, the STRIP intervention/control groups were combined. In the analyses, an interaction term for STRIP study group (intervention/control) × childhood parental smoking exposure was introduced to study whether the association between the childhood smoking exposure and the outcome was similar in the STRIP study groups. Both studies were conducted according to the guidelines of the Declaration of Helsinki and the study protocols have been approved by the local ethics committee. A written informed consent was provided by participants and/or their parents (or legal guardians) before the enrolment in the study.

### Procedures and measurements

#### Exposure variable parental smoking

Parents of the YFS participants self-reported their smoking habits (i.e. regular smoking lasting at least one year and current smoking status) at baseline visit in 1980 and in the first follow-up study in 1983. Parents who reported that they had ever smoked daily for at least one year were designated as ever smokers, while parents who indicated that they currently smoked either occasionally or daily were designated as current smokers. Combining the information on current and ever smoking, participants wereclassified into groups: 1) non-smoking parents(*N* = 901/29%), 2) onesmoking parent (*N* = 1478/47%) and 3) two smoking parents (*N* = 765/24%). The biological validity of the queried parental smoking data has been studied previously in the YFS data by comparing the parental responses of current and ever smoking with the offspring’s serum cotinine level [4]. As the number of smoking parents increased, also the cotinine level in the offspring increased.

Information on the current smoking status was queried from both STRIP parents at each study visit and used to define the parental smoking exposure in the present study. Two answers before and two answers after the participant’s age of 10 years was required to ensure the ever-smoking status during the follow-up. Those parents who reported smoking at least once during the study follow-up were considered as smokers. Similarly as in YFS, the STRIP participants were categorised according to reported parental smoking into three groups: 1) non-smoking parents (*N* = 428/54%), 2) onesmoking parent (*N* = 267/33%) and 3) two smoking parents (*N* = 102/13%) at the baseline.Additionally, inthe STRIP datachildhood exposure to parental smoking was further divided into: 1) early childhood exposure (7 months −2 years)(exposed *N* = 185, non-exposed *N* = 363), 2) childhood exposure (3–20 years)(exposed *N* = 232, non-exposed *N* = 302). *N* = 253 participants were exposed to parental smoking both in early childhood (7 months–2 years) and in later childhood (3–20 years).

#### Outcome variables and covariates

Weight and height have been measured at all study visits in both cohorts between 1989–2009/2010 in STRIP and 1980–2011/2012 in YFS. BMI was calculated as weight (kg)/height (m) [2]. Overweight/obesityand obesity was defined according to the Cole’s criteria [25] among children aged < 18 years, and as BMI ≥25kg/m^2^for overweight/obesity and ≥30kg/m^2^ for obesity among participants aged ≥18years.Waist circumference was measured in all YFS follow-up studies from the year 2001 (participants aged 24–39 years).In STRIP, waist circumference measurement was included in the study protocol from the year 1997 (participants aged 7 years). Central obesitywas defined as waist circumference > 90cm in women and >100cm in men [26] in YFS and as a waist-to-height ratio > 0.50 [27] in STRIP. Description of additional outcome variables for adiposity including skinfold and abdominal fat thicknesses are presented in the Supplementary Material. Measurement of covariates including educational level of the participants and the parents, participant’s own smoking, birth weight, dietary intake and physical activity as well as parental smoking during pregnancy (in STRIP), has been described in the Supplementary Material.

### Statistical analysis

Repeated Mixed Models and Generalised Estimating Equations were conducted to examine the associations between childhoodexposure to parental smoking and overweight/obesityfrom childhood to adulthood. All regression analyses were conducted as multivariable models including age and sexas covariates (Model 1). After that, the analyses were additionally adjusted for participant’s own or his/her parents’ educational level (Model 2). Finally, the analyses were additionally adjusted for participant’s own smoking status,birth weight, parental ages, longitudinal diet and physical activity(Model 3). In the STRIP, the analyses were conducted separately for the childhood exposure (3–20 years) and for the exposure during early childhood (7 months– 2 years). All multivariable model analyses were restricted to the participants with no missing data on childhood exposure to parental smoking, outcomes or any of the covariates (YFS: *N* = 2196 in the analyses for overweight/obesity and central obesity, *N* = 2303 in the analyses for adolescence skinfold measurements/thickness; STRIP childhood exposure (3–20 years):*N* = 537 in the analyses for overweight/obesity, *N* = 534 for central obesity, and *N* = 523 in the analyses for abdominalfatthickness; STRIP early childhood exposure (7 months–2years): *N* = 551 in the analyses for overweight/obesity, *N* = 548 for central obesity, and *N* = 537 in the analyses for abdominal fat thickness). Finally, in the STRIP data, sensitivity analyses were conducted excluding mothers (*N* = 73, 12%) and fathers (*N* = 143, 29%) who reported smoking during pregnancyor having stopped smoking prior to orduring pregnancy, and interaction between smoking exposure and intervention group was analysed. Non-normally distributed variables (i.e. variables indicating skinfold thickness in the YFS and abdominal fat thickness in the STRIP data) were corrected for skewness using logarithmic transformation. All statistical analyses were performed using SAS 9.4 and *p* < .05 was used as the level of significance.

## Results

Characteristics of the study population are presented in the Table 1.

**Table 1.** Characteristics of the study population among the participants exposed and non-exposed to parental smoking.

	Exposure to parental smoking during 3–21 years		Exposure to parental smoking during 3–20 years	
Participants	YFS		STRIP	
	Exposed *N* = 1512–1595	Non-exposed *N* = 642–673	Exposed *N* = 282	Non-exposed *N* = 350
Sex (women), %	55.3	55.9	50.4	49.1
Own smoking until the age of 20/ 21 years, %^a^	28.6 (1429)***	18.3 (618)***	26.2***	12.3***
Own smokingduring the follow-up in YFS, %^a^	58.3***	41.4***	–	–
In the latest study visit				
the prevalence of overweight/obesity, % (n)^b^	58.7 (759)*	52.1 (272)*	20.4 (35)	15.1 (39)
the prevalence of central obesity, % (n)^c^	36.6 (474)*	30.3 (159)*	17.0 (32)	9.5 (25)
Own educational level, %^d^	5.4 / 72.3 / 22.4***	3.4 / 66.0 / 30.5***	–	–
Birth weight, g	3506 (546)	3551 (497)	3547 (536)	3607 (491)
Maternal age, years^e^	26.3 (5.48)***	27.6 (5.41)***	29.8 (4.59)	30.2 (4.68)
Paternal age, years^e^	28.9 (6.50)**	29.8 (6.12)**	31.6 (5.81)	32.2 (5.37)
Parental educational level, %^d^	66.9 / 22.5 / 10.6*	62.6 / 22.7 / 14.6*	3.2 / 72.3 / 24.5***	2.6 / 53.1 / 44.3***

a Ever daily smoking during follow-up. Participants own smoking during 12–21 years in the YFS and 10–20 years in the STRIP.

b Body mass index ≥ 25kg/m^2^ for adults and according to the Cole in children; exposed *N* = 1292, non-exposed *N* = 522 in YFS; exposed *N* = 188, non-exposed *N* = 262 in STRIP.

c In women >90cm/in men >100cm/in children <18 years a waist circumference to height ratio >0.50; exposed *N* = 1292, non-exposed *N* = 522 in YFS; exposed *N* = 188 non-exposed *N* = 262 in STRIP.

d Comprehensive school/secondary or non-academic education/academic education. The participant’s own highest educational level queried in 2001, 2007 or 2011 (during ages 24–49 years) in the YFS. The highest educational level of either mother or father queried at the child’s age of 6–30 years (in 1983–1992, YFS) and at the age of 1, 5 or 9 years (in 1990–2001, STRIP).

e At participant’s birth values are means (standard deviations) for continuous variables and percentages for categorical variables. *P*-values are from T-tests or Qhi-Square tests (for educational level) Boldface indicates statistical significance (*p < .05, **p < .01, ***p < .001).

### Parental smoking exposure and offspring body mass index

Analyses in the YFS data showed that the participants with either one or two smoking parents had higher BMI compared to the participants with non-smoking parents (Supplementary Figure 3). Similar but non-significant associationwas seen in the STRIP data. Thus, the one or two smoking parents’ groups were combined for further analyses.

### Parental smoking exposure and the offspring risk of overweight/obesity

The longitudinal prevalence of overweight/obesity (BMI ≥ 25kg/m^2^) in both study cohorts is presented in the Supplementary Figure 4. In the YFS cohort, the age and sex adjusted risk for overweight/obesity was higher for those with at least one smoking parent compared to those with non-smoking parents over the whole study period(exposed *N* = 1522, non-exposed *n* = 647; Model 1: RR 1.16, 95% confidence interval (95%CI) 1.05–1.27; Table 2/Panel A). The point estimates remained essentially similar after additional adjustment for educational level, and after further adjustments for participant’s own smoking status, birth weight, parental ages, diet and physical activity. Additionally, we conducted analysis adjusted also for parental weight status and observed that the results remained essentially unchanged (data not shown).

**Table 2.** Exposure to parental smoking and the risk for overweight/obesity (Panel A), and central obesity (Panel B).

	Exposure to parental smoking during 3–21 years		Exposure to parental smoking during 3–20 years		Exposure to parental smoking prior to age 3 years	
	YFS		STRIP		STRIP	
*Panel A*						
Risk of overweight/obesity^d^	**RR** **aged 9–49 years (y)**	**95% CI** ^a^	**RR** **aged 3–20 y**	**95% CI** ^b^	**RR** **aged 2–20 y**	**95% CI** ^c^
Model 1	**1.16**	1.05–1.27**	**1.67**	1.17–2.39**	**1.51**	1.06–2.16*
Model 2	**1.15**	1.04–1.26**	**1.61**	1.11–2.34*	1.44	0.99**–**2.08
Model 3	**1.13**	1.02–1.24**	**1.57**	1.10–2.26*	1.38	0.96**–**1.97
*Panel B*						
Risk of central obesity^g^	**RR** **aged 24–49 y**	**95% CI** ^a^	**RR** **aged 7–20 y**	**95% CI** ^e^	**RR** **aged 7–20 y**	**95% CI** ^f^
Model 1	**1.26**	1.08–1.47**	**1.68**	1.12–2.51*	**1.86**	1.26–2.75**
Model 2	**1.22**	1.05–1.43*	**1.52**	1.02–2.25*	**1.67**	1.12–2.48*
Model 3	**1.18**	1.01–1.38*	1.45	0.98**–**2.15	**1.58**	1.06–2.33*

a Exposed *N* = 1522 and non-exposed *N* = 647.

b Exposed *N* = 233 and non-exposed *N* = 304.

c Exposed *N* = 186 and non-exposed *N* = 365.

d Body mass index ≥25 kg/m^2^.

e Exposed *N* = 232 and non-exposed *N* = 302.

f Exposed *N* = 185 and non-exposed *N* = 363.

g in YFS: waist circumference >100cm for men and >90cm for women; in STRIP: waist-to-height ratio > 0.50.

RR = Risk Ratio; 95% CI = Confidence Interval.

Model 1 is adjusted for age and sex.

Model 2 is adjusted additionally for family socioeconomic status (SES) and own SES since the age of 24 years in YFS.

Model 3 is adjusted additionally for birth weight, own smoking status, parental ages, diet and physical activity.

Boldface indicates statistical significance (*p < .05, **p < .01); analyses conducted by Generalised Estimating Equations.

Also in the STRIP, a higher risk of overweight/obesity was observed among participants with at least one smoking parent (exposed *N* = 233, non-exposed *N* = 304; Model 1: RR 1.67, 95%CI 1.17–2.39) (Table 2/Panel A). The association of parental smoking and the risk of overweight was similar between the study groups (intervention and control; interaction between smoking exposure and STRIP study group; *p* = .47).

We additionally analysed the association between early childhood parental smoking exposure (7 months–2 years) and the risk of overweight/obesity (BMI ≥ 25kg/m^2^) from childhood to adulthood in the STRIP cohort (Table 2/Panel A). An increased age and sex adjusted risk of overweight/obesity was observedfor the participants exposed to parental smoking in early childhood compared to the non-exposed peers. The associations wereobserved also after further adjustment for parental educational level and further for participant’s own smoking status, birth weight, parental ages, diet and physical activity.

To study the possible differences in the association between parental smoking exposure and overweight/obesity during different life stages, we conducted separate analyses for childhood, adolescence, young adulthood and mid-adulthood. When the risk of overweight/obesity was analysed in the separate life stages, the results were in line with our main results (Supplementary Table 1).

We also conducted the analyses using obesity (BMI > 30 kg/m^2^) as outcome and found that the association between smoking exposure and the risk of obesity was in line with the results between smoking exposure and the risk of overweight/obesity (Supplementary Table 2).

#### Risk of central obesity

The longitudinal prevalence of central obesity in both study cohorts is presented in the Supplementary Figure 5. In the YFS, the age and sex adjusted risk for central obesity was higher among participants with at least one smoking parent compared to those with non-smoking parents (exposed *N* = 1522, non-exposed *N* = 647; Model 1: RR 1.26, 95%CI 1.08–1.47; Table 2/Panel B). The estimate diminished 30% after additional adjustment for educational level, and after further adjustments for participant’s own smoking status, birth weight, parental ages, diet and physical activity.

Similarly in STRIP, a higher age and sex adjusted risk for central obesity was found for participants who had been exposed to parental smoking compared to the non-exposed peers (exposed *N* = 232, non-exposed *N* = 302; Model 1: RR 1.68, 95%CI 1.12–2.51; Table 2/Panel B). Similar results were observedwhen the model was additionally adjusted forparental educational level. When adjusted further for participant’s own smoking status, birth weight, parental ages, diet and physical activity the estimate diminished 33% (Model 3: RR 1.45,95%CI 0.98–2.15). Additionally, we conducted analysis adjusted also for parental weight status and observed that the results remained essentially unchanged (data not shown). There was no significant difference in the association of parental smoking exposure and the risk of central obesity between the study groups (intervention and control; interaction between smoking exposure and STRIP study group; *p* = .83).

Additionally, the analyses for the association between early childhood exposure to parental smoking (7 months–2 years) and the risk of central obesity in the STRIP showed that the risk for central obesity was higher for the exposed participants compared to their non-exposed peers (Table 2/Panel B).

For central obesity, the life stages specific analyses showed that the association of parental smoking exposure was evident already in childhood and became stronger towards adulthood (Supplementary Table 1).

#### Additional analyses for adiposity

The results of the additional analyses for childhood exposure to parental smoking and adiposity are presented in the Tables 3 and 4. (Supplementary Figures 6 and 7). In the YFS, the exposed participants had higher adiposity compared to the non-exposed participants. However in the STRIP, exposure to parental smoking was not associated with adiposity after full adjustments. There was no significant difference in the association of parental smoking exposure and the risk of adiposity between the study groups (intervention and control; interaction between smoking exposure and STRIP study group; *p* = .72/.91 for adiposity outcomes).

**Table 3.** Exposure to parental smoking during aged 3–21 years and skinfold thickness^a^ values in childhood and young adulthood.

YFS	Exposed *N* = 1621	Non-exposed *N* = 682
Subscapular (mm)^b^		
Model 1	11.0 (0.13)	10.5 (0.19)
Model 2	10.9 (0.15)	10.4 (0.20)
Model 3	11.0 (0.18)	10.5 (0.21)
Triceps (mm)^b^		
Model 1	11.4 (0.12)*	10.9 (0.17)*
Model 2	11.3 (0.13)*	10.8 (0.18)*
Model 3	11.2 (0.15)*	10.8 (0.19)*
Biceps (mm)^b^		
Model 1	6.4 (0.07)**	6.0 (0.10)**
Model 2	6.4 (0.08)**	6.0 (0.11)**
Model 3	6.3 (0.11)**	6.0 (0.13)**

a Measured by Harpenden callipers.

b Age 9–24 years.

Values are means (standard error).

Model 1 is adjusted for age and sex.

Model 2 is adjusted additionally for family socioeconomic status (SES and own SES since the age of 24 years in YFS.

Model 3 is adjusted additionally for own smoking status, birth weight, parental ages, diet and physical activity.

Boldface indicates statistical significance (*p < .05, **p < .01), analyses conducted for log-transformed values and p-values are from Repeated Mixed Model.

**Table 4.** Exposure to parental smoking and adolescence^a^ abdominal fat thickness (at xiphoid process/at navel) using ultrasound.

STRIP	Exposure to parental smoking during 3–20 years		Exposure to parental smoking prior to age of 3 years	
	Exposed *N* = 226	Non-exposed *N* = 297	Exposed *N* = 182	Non-exposed *N* = 355
**at xiphoid process (mm)**				
Model 1	**23.7 (0.50)**	22.3 (0.44)*	**24.1 (0.56)**	22.3 (0.40)*
Model 2	24.1 (0.85)	22.9 (0.78)	**24.5 (0.93)**	23.0 (0.76)*
Model 3	24.6 (0.88)	23.7 (0.84)	25.1 (0.95)	23.8 (0.81)
**at navel(mm)**				
Model 1	**21.7 (0.73)**	18.9 (0.64)*	**21.8 (0.82)**	19.1 (0.59)*
Model 2	21.4 (1.15)	20.3 (1.09)	22.7 (1.23)	20.5 (1.06)
Model 3	23.1 (1.18)	21.3 (1.16)	23.4 (1.25)	21.6 (1.12)

a Age 13, 15, 17, 19.

Values are means (standard error).

Model 1 is adjusted for age and sex.

Model 2 is adjusted additionally for family socioeconomic status (SES) and own SES since the age of 24 years in YFS.

Model 3 is adjusted additionally for own smoking status, birth weight, parental ages, diet and physical activity.

Boldface indicates statistical significance (*p < .05); analyses conducted for log-transformed values by Repeated Mixed Model.

### Sensitivity analyses

We conducted sensitivity analyses in the STRIP databy excluding the participants whose mother and father reported smokingduring pregnancy or having stopped smokingprior to or during pregnancy. These analyses are presented in Supplementary materials (Supplementary Table 3 and 4).

## Discussion

The present study showed that the individualsexposed to parental smoking in childhood/adolescence have an increased risk for life-course overweight/obesityand central obesity. Additionally, childhood exposure to parental smoking may associate with increased childhood and young adulthood adiposity measured by skinfold thickness. If these associations are causal, ournovel findings indicate that prevention of parental smoking would enhance not only the smokers own health but also lessen the risk of long-term overweight/obesityintheir offspring.

The aetiology of overweight and obesity is multifactorial. Evidently, the exposure to parental smoking is one of many factors contributing to the development of obesity. nergy-dense diet and physical inactivity are key lifestylefactors linked to overweight and obesity [28]. Moreover, the lifestyle factors adversely affecting weight status often tend to cluster [29–31], which complicates the determination of a specific factor affecting the risk of overweight and obesity. In this study, we considered a wide range of potential covariates that are linked to the risk of overweight/obesity. For instance, low socioeconomic status [32] as well as high [33] and low [34] birth weight have been associated with increased risk of overweight and obesity in childhood. In addition to individual’s own factors, parentalfactors have been suggested to affect the development of overweight and obesity in the offspring. For example, high maternal age at childbirth has been associated with increased body fat accumulation in the offspringduring chilhdood [35] and increased BMIduring adolescence [36]. Noticeably, own smoking may be a marker of the accumulation of unhealthy lifestyle factors and thus increase the risk of overweight [31]. However, active smoking may also reduce the weight of an individual [37]. Therefore, for example, statistical adjustment for own smoking may strengthen the association of childhood exposure to parental smoking and the risk of adulthood overweight/obesity.

In our analyses, the association of childhood smoking exposure and the risk of overweight/obesity assessed by BMI persisted even after taking account several covariates. Similarly, the association of smoking exposure and the risk of central obesity was seen in both cohorts, the YFS and the STRIP study. The effect of smoking exposure remained significant in the fully-adjusted models in the YFS, and was only marginally diluted when adjusted for covariates such as birth weight, parental ages, own smoking status, own or parental education, diet and physical activity. None of these covariates, however, diluted the effect when considered individually. Therefore, these covariates did not offer further insights into to mechanisms explaining the observed association between parental smoking and unfavourable weight development. To confirm the differing findings in central obesity in the YFS (childhood and adulthood) and the STRIP (childhood and adolescence) cohorts, we examined the association of smoking exposure with childhood and young adulthood skinfold thickness in the YFS, and with childhood/adolescence abdominal fat in the STRIP. Using these outcomes, we found that the exposed YFS participants had thicker skinfolds (triceps and biceps) in childhood/adolescence than the non-exposed YFS participants. Similarly, the association between parental smoking exposure and increased abdominal fat thickness measured by ultrasound was observed in the STRIP population. The effect of smoking exposure on abdominal fat thickness was diluted to statistically non-significant in fully adjusted models. This finding may reflect the reduction of statistical power in the fully adjusted model, as none of the variables diluted the effect when considered individually. Nevertheless, in general the ultrasound data showed similar trends than other adiposity outcomes used in this study. The further analyses in the STRIP showed that the association between early (between ages 7 months and 2 years) smoking exposure and risk of central obesity as well as abdominal fat measured by ultrasound was similar to the association between childhood and adolescence (3–20 years) smoking exposure and the same obesity related measurements. Our results thus suggest that the duration of the smoking exposure may not be the most meaningful factor underlying the link between the smoking exposure and the overweight/obesity outcome.

Previous studies have shown that maternal smoking during pregnancy may be associated with increased BMI and waist circumference in childhood and adulthood [11–14], which means that prenatal exposure might confound also our results. Therefore, using the STRIP data we conducted sensitivity analyses in which we excluded those mothersand fathers who reported smokingduring pregnancy or having stopped smokingprior to or during pregnancy from the analyses. The results from the sensitivity analysesshowed that the association between childhood smoking exposure and the risk of central obesity and adiposity measures is dilutedafter such exclusions. Noticeably, over 80% of those parents who reported smoking during pregnancy or having stopped smoking prior to or during pregnancy continued smoking after pregnancy. Therefore, the size of the study population, especially the number of exposed participants, decreased substantially in the sensitivity analyses. Thus, the observed dilution of the associations in the sensitivity analyses may reflect reduced statistical power. Concluding, we are not able to fully distinguish the impact of prenatal smoking exposure on offspring weight from the childhood exposure. However, even though the associations shown in the present study may partly reflect the effect of prenatal parental smoking exposure, our study is the first to show the adverse associations of parental smoking on offspring adiposity development from early childhood to midlife.

The biological links between second-hand smoking exposure in childhood and offspring obesity are still unclear. A previous study in rats has shown that exposure to tobacco smoke between postnatal days 3 and 21, especially during lactation associates with increased BMI and central obesity in adulthood [21,22]. The mechanism between smoking exposure and weight development is unknown but it is suggested that smoking exposure causes hormonal and metabolic dysfunction. It is suggested that serum leptin hormoneconcentration increases in those who are exposed to smoking during lactation [38] causing leptin resistance [22] and may thus cause positive energy balance and overweight.

### Strengths and limitations of this study

The major strength of this study is the exceptionally long follow-up of bothcohorts; the STRIP participants have been followed-up for 20 years while the follow-up time of the YFS cohort exceeds to over 30 years. During the extensive follow-up times both cohorts have followed their participants from infancy/childhood to adulthood which enables the adoption of a life-course approach to the studied associations. Additional strength in the YFS and STRIP studies isthe large number of participants that have remained within the study. In the STRIP, a major advantage is the biannual/annual study visits throughout the first 20 years of life.

There are also some limitations in the present study that need to be discussed. First, smoking status was self-reported which may cause underestimation of the smoking habits [39]. This means that those parents who reported to be smokers probably are smokers whereas those who reported to be non-smokers might also be smokers. Thus, the possible bias due to misreporting can be assumed to dilute rather than overestimate the true associations. Second, measurement of parental smoking was not identical in both cohorts. In the YFS, smoking status was askedtwice including the questions of ever and current smoking. Simultaneously in the STRIP, current smoking status was askedrepeatedlyusing a question with dichotomy scale (yes/no), and at least four answers with at least one indicating smokingwere required to be classified as smoker. Therefore, the accuracy of the smoking status queried in the YFS might be somewhat weaker than in the STRIP cohort possibly causing discrepancy in the results. Third, maternal smoking during pregnancy was not measured in the YFS cohort, and therefore, we were able to take prenatal smoking exposure into account only in the STRIP data.

## Conclusions

Exposure to parental smoking in childhood was associated with increased risk of overweight/obesity, central obesity and adiposity measured by skinfold thickness from childhood to adulthood. These findings highlight the importance of promotion of parental smoking cessation throughout their offspring’s childhood and adolescence as it might translate into healthy weight development trajectory among their children.

## Supplementary Material

Supplemental Material
