# Supplementary material for: Childhood exposure to parental smoking and life-course overweight and central obesity
Source: Ann Med. 2021 Feb 22;53(1):208–16. doi: 10.1080/07853890.2020.1853215 (PMC7901689; doi:10.1080/07853890.2020.1853215)
Supplement: Supplemental Material [file IANN_A_1853215_SM1082.zip › Supplementary_figures_revised.docx]

**
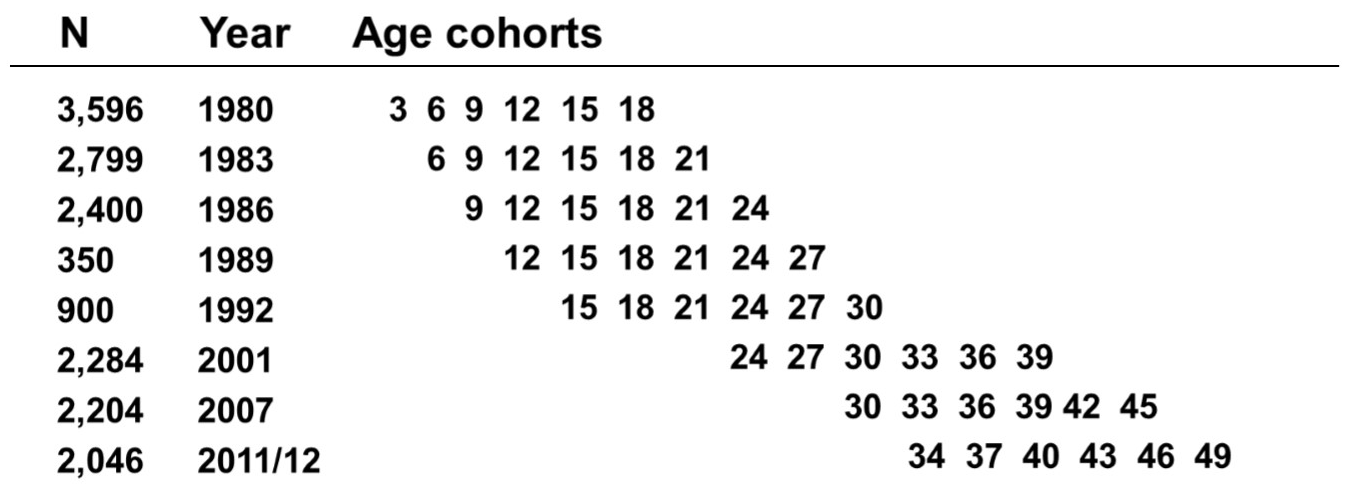
**

**Supplementary Figure 1.** Study design of YFS.

Turku city well-baby clinics, 1990-1992

1880 infants aged 5 months

1116 infants aged 7 months

Randomization

Control group, N=522

Intervention group, N=564

At 20 years, N=468

**Supplementary Figure 2.** Study design of STRIP.


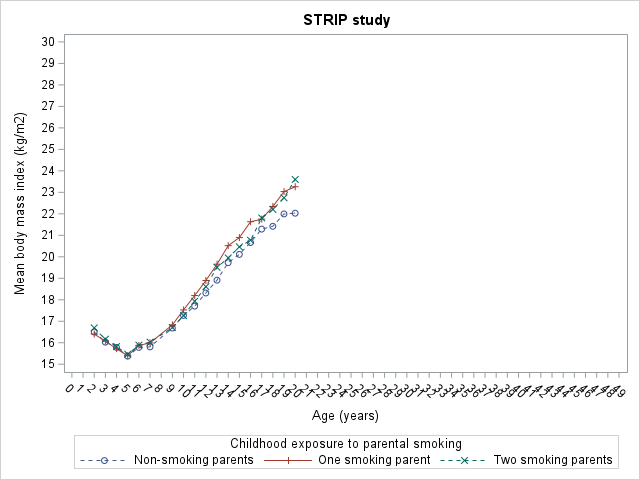

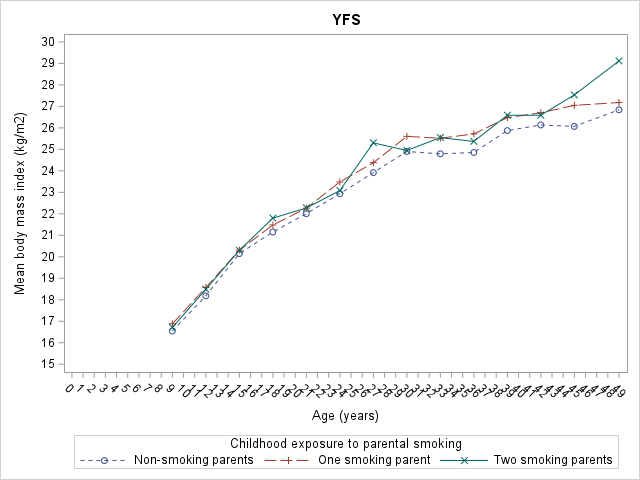


non-smoking parents vs one smoking parent: β=0.38; SE= 0.16; P=0.02

non-smoking parents vs two smoking parents: β=0.54; SE=0.19; P=0.006

one smoking parent vs two smoking parents: β=0.16; SE=0.17; P=0.36

non-smoking parents vs one smoking parent: β=0.56; SE=30; P=0.11

non-smoking parents vs two smoking parents: β=0.32; SE=0.45; P=0.76

one smoking parent vs two smoking parents: β=0.24; SE=0.46; P=0.46

**Supplementary Figure 3.** Longitudinal body mass index (BMI) trajectories among YFS and STRIP cohort participants according to parental smoking exposure. The mean BMI trajectories was the lowest in the YFS participants with non-smoking parents and the highest in the participants with two smoking parents (N=2303, P^[[1]](#footnote-1)^=0.012; adjusted for age, sex, own smoking status, socioeconomic status, birth weight, parental ages, diet and physical activity). In the STRIP participants, the mean BMI was the lowest in those with non-smoking parents but difference in mean BMI between exposure groups was non-significant (N=538, P^1^=0.28).


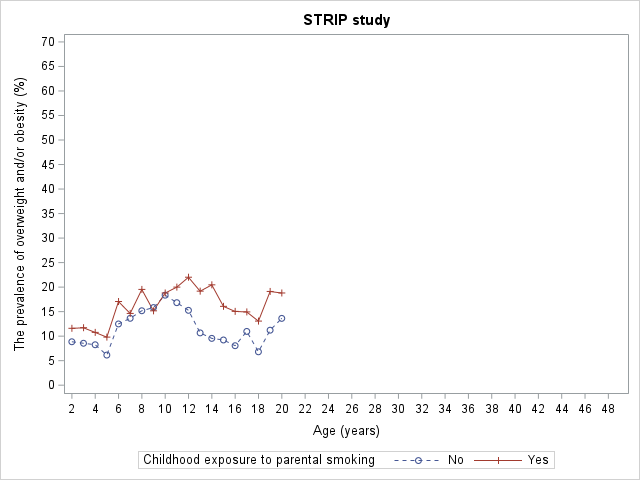

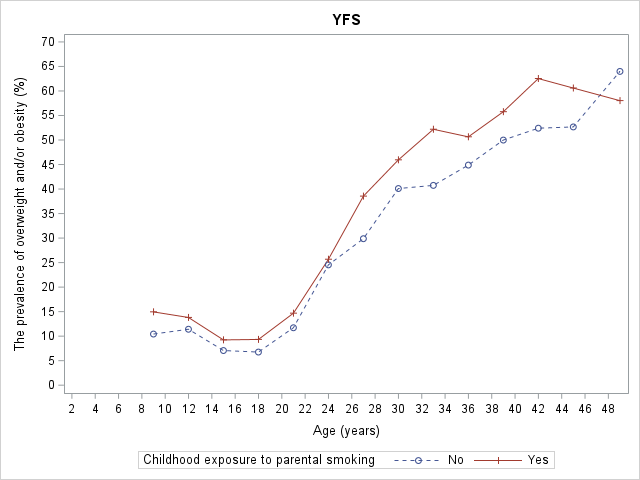


**Supplementary Figure 4.** The longitudinal prevalence of overweight and/or obesity (BMI ≥25kg/m^2^) was higher for those with at least one smoking parent compared to those with non-smoking parents in the YFS and STRIP (YFS: N=2169; RR 1.13, CI 1.02–1.24, P^[[2]](#footnote-2)^=0.014; STRIP: N=537; RR 1.57, CI 1.10–2.26, P^2^=0.015, when adjusted for age, sex, socioeconomic status, own smoking, birth weight, parental ages, diet and physical activity).


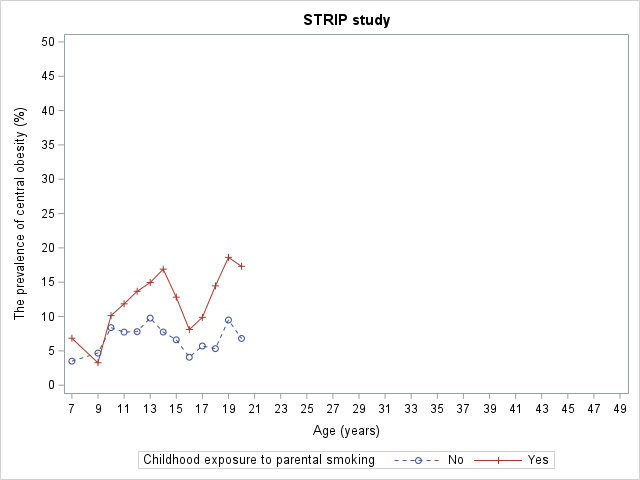

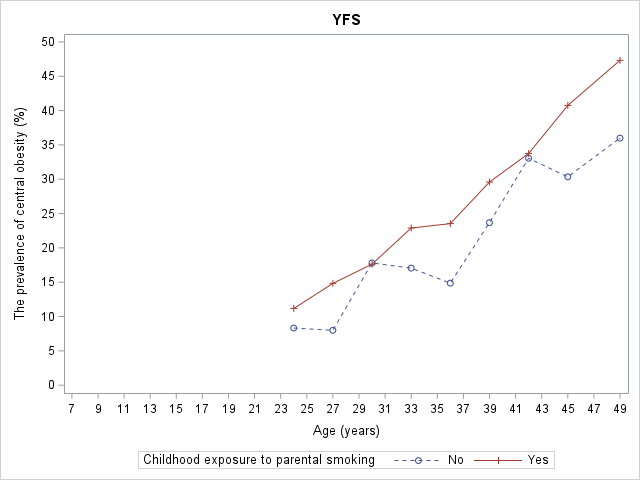
**Supplementary Figure 5.** The longitudinal prevalence of central obesity (in the YFS: waist circumference >100cm for men and >90cm for women; in the STRIP: waist-to-height ratio>0.50) among the participants exposed parental smoking and those non-exposed. (YFS: N=2169, RR 1.18, 95%CI 1.01–1.38, p=0.037^[[3]](#footnote-3)^; STRIP: N=534; RR 1.45 95%CI 0.98–2.15, P=0.066^3^, adjusted for age, sex, socioeconomic status, own smoking, birth weight, parental ages, diet and physical activity.)


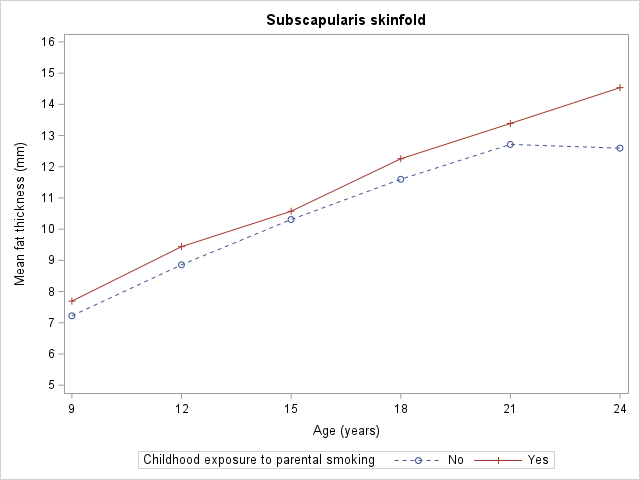

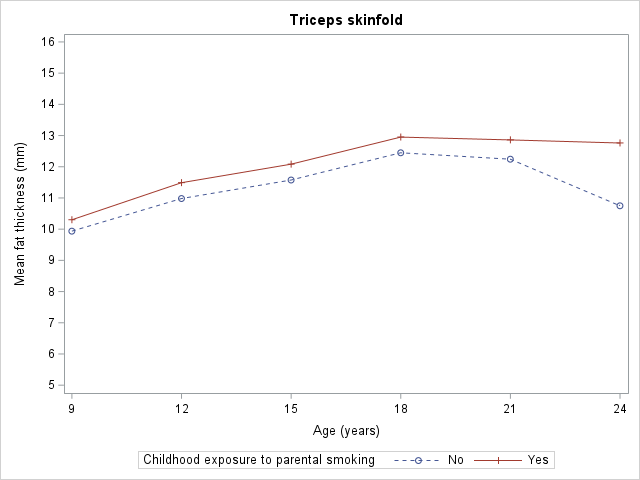

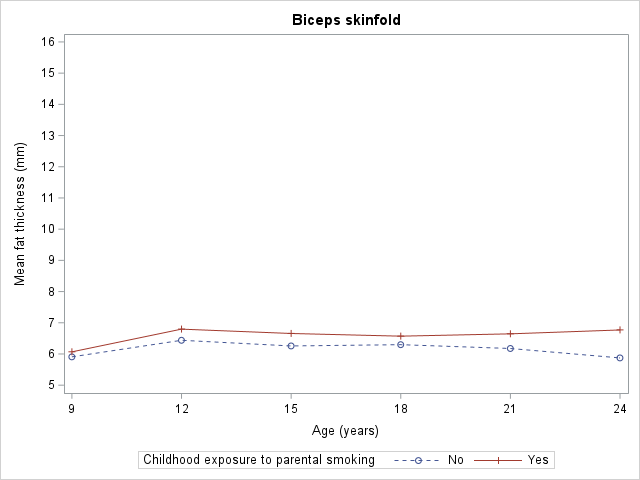


**Supplementary Figure 6.** In the YFS, the mean skinfold (subscapularis, triceps and biceps) thickness (mm) trajectories during 9–24 years in two groups: participants exposed (N=1621) or non-exposed (N=682) to parental smoking in childhood. For subscapularis: exposed mean=11.0mm (SE 0.18) vs non-exposed mean=10.5mm (SE 0.21), P=0.12^[[4]](#footnote-4)^; for triceps: exposed mean=11.2mm (SE 0.15) vs non-exposed mean=10.8mm (SE 0.19), P=0.03^4^ and for biceps: exposed mean=6.3mm (SE 0.11) vs non-exposed mean=6.0mm (SE 0.13), P=0.007^4^ when adjusted for age, sex, socioeconomic status, own smoking, birth weight, parental ages, diet and physical activity.


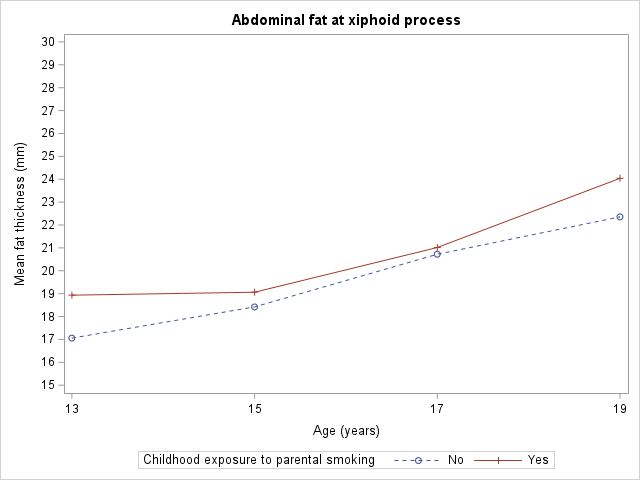

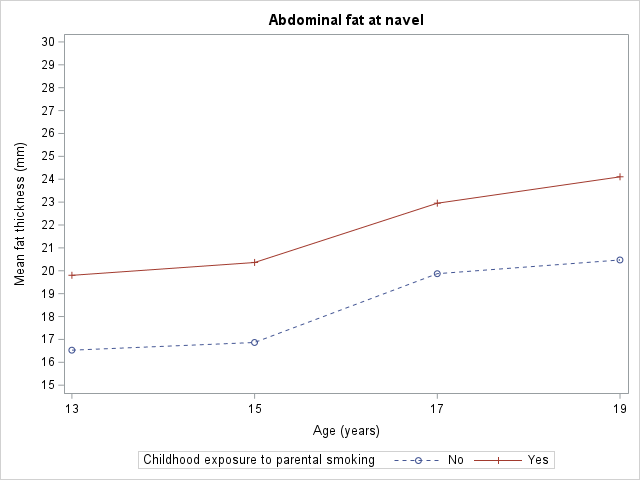


**Supplementary Figure 7**. In the STRIP, the mean abdominal fat thickness (mm) (at xiphoid process and at navel) trajectories measured by ultrasound in adolescence in two groups: participants exposed (N=226) or not (N=297) to parental smoking in childhood. At xiphoid process: exposed mean=24.6mm (SE 0.88) vs. non-exposed mean=23.7 (SE 0.84), P=0.23^[[5]](#footnote-5)^. At navel: exposed mean=23.1 (SE 1.18) vs. non-exposed mean=21.3 (SE 1.16), P=0.25^5^; when adjusted for age, sex, socioeconomic status, own smoking status, birth weight, parental ages, diet and physical activity.

1. P-values are from Repeated Mixed Model [↑](#footnote-ref-1)
2. P-values are from Generalized Estimating Equations [↑](#footnote-ref-2)
3. P-values are from Generalized Estimating Equations [↑](#footnote-ref-3)
4. Analyses conducted for log-transformed values and P-values are from Repeated Mixed Model [↑](#footnote-ref-4)
5. Analyses conducted for log-transformed values and P-values are from Repeated Mixed Model [↑](#footnote-ref-5)
