# Supplementary material for: Childhood exposure to parental smoking and life-course overweight and central obesity
Source: Ann Med. 2021 Feb 22;53(1):208–16. doi: 10.1080/07853890.2020.1853215 (PMC7901689; doi:10.1080/07853890.2020.1853215)
Supplement: Supplemental Material [file IANN_A_1853215_SM1082.zip › Supplementary_methods.docx]

**SUPPLEMENTARY MATERIAL**

**Childhood exposure to parental smoking and life-course overweight and central obesity**

**By Jaakkola JM et al.**

**Supplementary methods**

***Additional outcome variables for adiposity***

Adiposity was measured as skinfold thickness in the YFS and abdominal fat thickness in the STRIP. In the YFS, skinfold thickness was measured from triceps, biceps and subscapularis using Harpenden calipers (Holtain and Bull-British Indicators instruments) to 0.2-mm readings in the YFS follow-up visits in 1980, 1983, and 1986 (participants aged 3–24 years). In the STRIP, abdominal fat thickness was measured from ultrasound images as the sum of subcutaneous and pre-peritoneal fat thicknesses at xiphoid process and navel along the linea alba^1^. The measurement for abdominal fat thickness was biennial from the year 2002 (participants aged 13, 15, 17 and 19 years).

***Covariates***

In both studies, information on participant’s parents’ as well as participant’s own educational level was queried and used as an indicator or socioeconomic status (SES) in the present study. Information on educational level was categorized into: 1) comprehensive school, 2) secondary or non-academic education, and 3) academic education. In the YFS, information on educational level was queried in the follow-up studies in 1983, 1986, 1989 and 1992 (participants aged 6–30 years) from both parents and the highest educational level (either from the mother or the father) was selected to represent the family SES. In the case of missing information from either of the parents, the one obtained was used. Participant’s own educational level was queried in the follow-up studies in 2001, 2007 and 2011 (participants aged 24–49 years), and the most recent information was used. In the STRIP, participant’s childhood family SES was defined using the information on parental educational level queried at the study visits when the child was aged 1, 5 and 9 years (the follow-up studies in 1990–1991, 1995–1996 and 1998–1999) and categorized similarly to the YFS data. Again, the highest educational level either from mother or father was used, and the most recent available parental education information was considered. In the statistical analyses for the YFS data, parental educational level was used as an indicator of family SES until the participant’s age of 24 years, and the participant’s own educational level thereafter, while in the analyses for the STRIP data, parental educational level was used.

Participants’ *own smoking* habit (i.e. ever daily smoking) was assessed with a questionnaire in all follow-up studies from the age 12 years in the YFS, and from the age 10 years in the STRIP. The participants who reported ever smoking in any of the follow-up visits were considered as smokers. Participant’s *birth weight* was queried from the parents in the YFS and collected from the delivery hospital and well-baby clinic records in the STRIP. Data on *maternal and paternal smoking* status prior to or during pregnancy was drawn from the delivery hospital and well-baby clinic records in the STRIP. In both studies, mother’s and father’s age at the time of the participant’s birth was queried at the baseline study.

In the YFS, *dietary intake* was assessed using 48 h dietary recall in the follow-up visits 1980, 1983, 1986, 1989 and 2001 (participants aged 3–39 years) whereas food frequency questionnaire in the follow-up visits in 2007 and 2011 (participants aged 30–49 years). A dietary intake index was defined based on three longitudinally available food groups: 1) vegetables, fruit and berries, 2) fish and 3) sugared beverages. The intake of vegetables, fruit and berries at least once a day, fish more than once a week and sugared beverages twice or less a week yielded one point each. The index was calculated summarizing the three food groups (range 0–3).

In the STRIP, dietary intake was assessed annually at each study visits (at the age of 8 and 13 months, and between 2 and 20 years) using a food record. Diet score was defined based on intakes of 11 food groups.^2^ Fiber-rich grains, fish, fruits and berries, vegetables, nuts and seeds, low-fat unsweetened dairy products and vegetable-oil based fats were designated as favorable foods, whereas red and processed meat, desserts, sugared beverages and salty snacks were designated as unfavorable foods. Intake of each food group (as grams/ day) were categorized into quartiles and assigned ascending values (0, 1, 2, 3) for favorable foods and descending values (3, 2, 1, 0) for unfavorable foods. These values were summed to generate the diet score (range, 0–33 points), with higher scores representing healthier diet. The diet score was used as a covariate longitudinally in the analysis.

*Physical activity* was assessed through a self-administered questionnaire in all study visits from year 1980 and from the age of 9 years in the YFS and biennially at the age of 13, 15, 17 and 19 years (from the study visits in 2002) in the STRIP. Physical activity index was defined in the YFS^3^ and the index was used as covariate longitudinally in the analysis. In STRIP, to describe physical activity level, metabolic equivalent rate (MET) index was calculated by multiplying the frequency, mean duration in minutes, and mean intensity of weekly leisure-time physical activity as described previously.^4^ The index indicates the multiple of the resting metabolic rate (MET; h/wk) and was used longitudinally as a covariate.

**References**

1. Suzuki R, Watanabe S, Hirai Y, et al. Abdominal wall fat index, estimated by ultrasonography, for assessment of the ratio of visceral fat to subcutaneous fat in the abdomen. *Am J Med*. 1993;95(3):309-314.

2. Matthews LA, Rovio SP, Jaakkola JM, et al. Longitudinal effect of 20-year infancy-onset dietary intervention on food consumption and nutrient intake: The randomized controlled STRIP study. *Eur J Clin Nutr*. 2018.

3. Telama R, Viikari J, Valimaki I, et al. Atherosclerosis precursors in Finnish children and adolescents. X. leisure-time physical activity. *Acta Paediatr Scand Suppl*. 1985;318:169-180.

4. Raitakari OT, Taimela S, Porkka KV, et al. Patterns of intense physical activity among 15- to 30-year-old Finns. the cardiovascular risk in Young Finns Study. *Scand J Med Sci Sports*. 1996;6(1):36-39.
