# Supplementary material for: Childhood exposure to parental smoking and life-course overweight and central obesity
Source: Ann Med. 2021 Feb 22;53(1):208–16. doi: 10.1080/07853890.2020.1853215 (PMC7901689; doi:10.1080/07853890.2020.1853215)
Supplement: Supplemental Material [file IANN_A_1853215_SM1082.zip › Supplementary_tables_revised.docx]

**Supplementary Table 1.** Childhood exposure to parental smoking and the risk of overweight/obesity and central obesity divided the outcomes into life stages, e.g. childhood, adolescence, young adulthood, and mid-adulthood.

| **YFS** | **Exposure between the ages 3–21 years** | | | | | | | |
| --- | --- | --- | --- | --- | --- | --- | --- | --- |
|  |  | | adolescence and young adulthood  (12-21 y) (n=2085) | | | | mid-adulthood  (24–49 y) (n=2034) | |
| Risk of overweight/obesity |  |  | **RR** | **95% CI** |  |  | **RR** | **95% CI** |
|  |  |  | 1.41 | 0.90–2.19 |  |  | 1.12 | 1.02–1.23* |
| Risk of central obesity |  |  | **RR** | **95% CI** |  |  | **RR** | **95% CI** |
|  |  |  | - | - |  |  | 1.18 | 1.00–1.38* |
| **STRIP** | **Exposure between the ages 3–20 years** | | | | | | | |
| Risk of overweight/obesity | childhood (3-11 y) (n=596) | | adolescence (12-17 y) (n=512) | | young adulthood (18-20 y)  (n=407) | |  | |
|  | **RR**^a^ | **95% CI** | **RR** | **95% CI** | **RR**^a^ | **95% CI** |  |  |
|  | 1.10 | 0.90–1.33 | 1.50 | 1.09–2.05* | 1.37 | 0.93–2.03 |  |  |
| Risk of central obesity | childhood (7-11 y)  (n=580)^1^ | | adolescence (12-17 y) (n=509) | | young adulthood (18-20 y)  (n=418) | |  |  |
|  |  |  | **RR** | **95% CI** | **RR**^a^ | **95% CI** |  |  |
|  |  |  | 1.40 | 0.91–2.15 | 1.91 | 1.19–3.07** |  |  |

^a^ excluding physical activity, measured from at age of 13,15,17,19 years in STRIP and since the age of 9 years in YFS

RR=Risk Ratio, 95%CI= 95% Confidence Interval

Boldface indicates statistical significance (*p<0.05, **p<0.01)

Analyses conducted by Generalized Estimating Equations; adjusted for age, sex, family socioeconomic status (SES) and own SES since the age of 24 years in YFS, birth weight, own smoking status, parental ages, diet and physical activity

**Supplementary Table 2.** Childhood exposure to parental smoking and the risk of obesity in the YFS and STRIP.

|  | **Exposure to parental smoking during 3–21 years (y)** | | **Exposure to parental smoking during 3–20 y** | |
| --- | --- | --- | --- | --- |
|  | **YFS** |  | **STRIP** |  |
|  | **RR**^a^ | **95% CI** | **RR**^b^ | **95% CI** |
| **Risk of obesity^c^** | aged 9–49 y |  | aged 2–20 y |  |
| Model 1 | **1.33** | **1.06 – 1.67**** | **1.71** | **1.06 – 2.77*** |
| Model 2 | **1.31** | **1.04 – 1.65*** | **1.61** | **0.99 – 2.64*** |
| Model 3 | **1.27** | **1.01 – 1.60*** | **1.61** | **0.98 – 2.65*** |

^a^ Exposed N=1522 and non-exposed N=647

^b^ Exposed N=233 and non-exposed N=304

^c^ Body mass index ≥30 kg/m^2^ in women and men, according to the Cole in children

RR=Risk Ratio; 95%CI=95% Confidence Interval

Model 1 is adjusted for age and sex

Model 2 is adjusted additionally for family socioeconomic status (SES) and own SES since the age of 24 years in YFS

Model 3 is adjusted additionally for birth weight, own smoking status, parental ages, diet and physical activity

Boldface indicates statistical significance (*p<0.05, **p<0.01); analyses conducted by Generalized Estimating Equations;

**Supplementary Table 3.** Exposure to parental smoking (in childhood/early childhood) and the risk of overweight/obesity and central obesity

|  | **Exposure to parental smoking during 3–20 years** | | | | **Exposure to parental smoking prior to age 3 years** | | | |
| --- | --- | --- | --- | --- | --- | --- | --- | --- |
|  | Excluded^a^ smoking mothers (N=63) | | Excluded^a^ smoking fathers (N=182) | | Excluded^a^ smoking mothers (N=66) | | Excluded^a^ smoking fathers (N=189) | |
|  | **RR** | **95% CI** | **RR** | **95% CI** | **RR** | **95% CI** | **RR** | **95% CI** |
| **Risk of overweight and/or obesity**^b^ | **during the follow-up**^c^ | | **during the follow-up**^d^ | | **during the follow-up**^e^ | | **during the follow-up**^f^ | |
| Model 1 | **1.60** | **1.09–2.35*** | 1.70 | 0.97–2.97 | 1.42 | 0.96–2.11 | 0.89 | 0.30–2.71 |
| Model 2 | **1.58** | **1.06–2.35*** | 1.67 | 0.95–2.93 | 1.37 | 0.90–2.08 | 0.90 | 0.29–2.75 |
| Model 3 | **1.48** | **1.01–2.19*** | 1.61 | 0.91–2.85 | 1.30 | 0.87–1.95 | 0.85 | 0.28–2.58 |
| **central obesity**^g^ | **during the follow-up**^h^ | | **during the follow-up**^i^ | | **during the follow-up**^j^ | | **during the follow-up**^k^ | |
| Model 1 | 1.49 | 0.96–2.33 | 1.32 | 0.67–2.57 | **1.70** | **1.09–2.63*** | 1.10 | 0.44–2.74 |
| Model 2 | 1.39 | 0.90–2.16 | 1.25 | 0.66–2.39 | 1.55 | 0.98–2.45 | 1.07 | 0.43–2.65 |
| Model 3 | 1.27 | 0.82–1.97 | 1.16 | 0.57–2.36 | 1.44 | 0.92–2.25 | 1.02 | 0.38–2.75 |

in STRIP.

^a^ Those who reported smoking during pregnancy or having stopped smoking prior to or during pregnancy

^b^ Body mass index≥25kg/m^2^

^c^ Age 2–20 years; exposed N=182 and non-exposed N=292

^d^ Age 2–20 years; exposed N=67 and non-exposed N=225

^e^ Age 2–20 years; exposed N=137 and non-exposed N=348

^f^ Age 2–20 years; exposed N=24 and non-exposed N=272

^g^ Waist-to-height ratio>0.50

^h^ Age 7–20 years; exposed N=181 and non-exposed N=290

^i^ Age 7–20 years; exposed N=67 and non-exposed N=224

^j^ Age 7–20 years; exposed N=136 and non-exposed N=346

^k^ Age 7–20 years; exposed N=24 and non-exposed N=271

RR=Risk Ratio; 95%CI=Confidence Interval

Model 1 is adjusted for age and sex

Model 2 is adjusted additionally for family socioeconomic status (SES) and own SES since the age of 24 years in YFS

Model 3 is adjusted additionally for birth weight, own smoking status, parental ages, diet and physical activity

Boldface indicates statistical significance (*p<0.05); analyses conducted by Generalized Estimating Equations

**Supplementary Table 4.** The association of exposure to parental smoking and adolescence^a^ abdominal fat thickness measured at xiphoid process and at navel using ultrasound in STRIP.

| **Skinfold thickness** | **Exposure to parental smoking during 3–20 years** | | | | **Exposure to parental smoking prior to age 3 years** | | | |
| --- | --- | --- | --- | --- | --- | --- | --- | --- |
|  | Excluded^b^ smoking mothers (N=63) | | Excluded^b^ smoking fathers (N=175) | | Excluded^b^ smoking mothers (N=52) | | Excluded^b^ smoking fathers (N=182) | |
|  | **Exposed N=175** | **Non-exposed N=285** | **Exposed N=66** | **Non-exposed N=219** | **Exposed N=133** | **Non-exposed N=338** | **Exposed N=24** | **Non-exposed N=265** |
| **at xiphoid process** |  | |  | |  | |  | |
| Model 1 | 23.7 (0.57) | 22.3 (0.45) | 23.1 (0.88) | 21.9 (0.49) | **24.1 (0.66)*** | **22.4 (0.42)*** | 22.7 (1.46) | 22.1 (0.44) |
| Model 2 | 24.1 (0.85) | 22.9 (0.78) | 24.5 (1.37) | 23.4 (1.15) | 24.5 (0.93) | 23.0 (0.76) | 24.2 (1.84) | 23.6 (1.13) |
| Model 3 | 24.6 (0.88) | 23.7 (0.84) | 25.2 (1.43) | 24.3 (1.28) | 25.0 (0.95) | 23.8 (0.81) | 24.6 (1.83) | 24.6 (1.24) |
| **at navel** |  | |  | |  | |  | |
| Model 1 | 21.4 (0.83) | 19.0 (0.65) | 20.4 (1.23) | 18.3 (0.68) | 21.8 (0.96) | 19.3 (0.60) | 19.3 (2.07) | 18.7 (0.62) |
| Model 2 | 22.4 (1.23) | 20.3 (1.12) | 23.8 (1.91) | 21.9 (1.60) | 22.8 (1.35) | 20.6 (1.10) | 22.8 (2.57) | 22.3 (1.58) |
| Model 3 | 23.2 (1.26) | 21.6 (1.21) | 24.4 (1.99) | 22.6 (1.77) | 23.5 (1.37) | 21.8 (1.18) | 23.1 (2.55) | 23.2 (1.74) |

^a^ Age 13, 15, 17, 19 years

^b^ Those who reported smoking during pregnancy or having stopped smoking prior to or during pregnancy

Values are means (standard error)

Model 1 is adjusted for age and sex

Model 2 is adjusted additionally for family socioeconomic status (SES and own SES since the age of 24 years in YFS

Model 3 is adjusted additionally for own smoking status, birth weight, parental ages, diet and physical activity.

Boldface indicates statistical significance (*p<0.05), analyses conducted for log-transformed values by Repeated Mixed Model
